# Circulating tight-junction proteins are potential biomarkers for blood–brain barrier function in a model of neonatal hypoxic/ischemic brain injury

**DOI:** 10.1186/s12987-021-00240-9

**Published:** 2021-02-10

**Authors:** E. Axel Andersson, Carina Mallard, C. Joakim Ek

**Affiliations:** Institute of Neuroscience and Physiology, Sahlgrenska Academy, University of Gothenburg, Medicinaregatan 11, 413 90 Gothenburg, Sweden

**Keywords:** Biomarkers, Blood–brain barrier, Hypoxia/ischemia, Neonatal, Tight-junctions

## Abstract

**Background:**

Neonatal encephalopathy often leads to lifelong disabilities with limited treatments currently available. The brain vasculature is an important factor in many neonatal neurological disorders but there is a lack of diagnostic tools to evaluate the brain vascular dysfunction of neonates in the clinical setting. Measurement of blood–brain barrier tight-junction (TJ) proteins have shown promise as biomarkers for brain injury in the adult. Here we tested the biomarker potential of tight-junctions in the context of neonatal brain injury.

**Methods:**

The levels of TJ-proteins (occluding, claudin-5, and zonula occludens protein 1) in both blood plasma and cerebrospinal fluid (CSF) as well as blood–brain barrier function via ^14^C-sucrose (342 Da) and Evans blue extravasation were measured in a hypoxia/ischemia brain-injury model in neonatal rats.

**Results:**

Time-dependent changes of occludin and claudin-5 levels could be measured in blood and CSF after hypoxia/ischemia with males generally having higher levels than females. The levels of claudin-5 in CSF correlated with the severity of the brain injury at 24 h post- hypoxia/ischemia. Simultaneously, we detected early increase in blood–brain barrier-permeability at 6 and 24 h after hypoxia/ischemia.

**Conclusions:**

Levels of circulating claudin-5 and occludin are increased after hypoxic/ischemic brain injuries and blood–brain barrier-impairment and have promise as early biomarkers for cerebral vascular dysfunction and as a tool for risk assessment of neonatal brain injuries.

## Background

Neonatal encephalopathy is a syndrome characterised by neurological dysfunction presenting as e.g. seizures and respiratory difficulties [1, 2]. Neonates that are diagnosed with neonatal encephalopathy (NE) are, if they survive, at high risk of developing life-long and permanent neurodevelopmental disabilities [3]. Although hypothermia has been shown to be a beneficial treatment under certain circumstances, since it has to be initiated within six hours and can only be implemented in some of the neonates, with a number needed to treat of around eight, novel or adjunctive treatments are needed [4, 5]. A key step in the development of new treatments is diagnostic tools enabling early diagnosis and/or monitoring of injury progression. The aetiology underlying brain injury in the newborn is complex and is likely to involve many factors, making early diagnosis very difficult [6]. The most commonly used early clinical tools to assess neonatal NE include cord blood gas analyses [7], Apgar and Sarnat scoring systems [8]. However, these methods have a low predictive value for the subsequent brain injury [9]. The resulting brain injuries are typically visualised by advanced imaging methods such as MRI and CT-scans [10], techniques that are both expensive and have a relatively low availability, in terms of both equipment and skilled personnel, in many countries [11]. This is important to note as it has been estimated that as many as 96% of neonates affected by NE are born in low- and middle-income countries [12]. There is a clinical need for better and affordable diagnostics of neonatal brain injuries that would enable early risk assessment and intervention as well as monitoring of injury development. Putative biomarkers for neuronal damage following NE would be a valuable diagnostic tool to predict long-term outcomes but has the drawback that they are usually measurable only after the injury has manifested [13].

The brain vasculature is a central factor in many human neonatal neurological disorders, such as intracranial haemorrhages and neonatal stroke [14], and the blood–brain barrier (BBB) is disrupted early in the disease process [15–20]. A biomarker that reflects the state and function of the neonatal brain vasculature could be a powerful predictor for brain injury, considering that studies in rats have shown that the dysfunction of the BBB mirrors the severity of hypoxic/ischemic (HI)-injuries [15, 16]. Additionally, assessment of BBB function is of importance for the success of treatments that are directed at the brain. The capillary endothelial cells of the cerebrovasculature are connected by continuous complexes of tight-junction (TJ) proteins, which restrict passage between the brain and the bloodstream, thus maintaining the integrity of the BBB and function of the brain [21]. Previous studies in adults have shown that TJ proteins are shed from the BBB and cerebral vasculature and enters the circulation in a model of adult stroke in rats [22], in humans following stroke [23] as well as after intracranial haemorrhage [24]. Furthermore, studies in cultured cerebral endothelial cells and in vivo adult rats have shown that the BBB is dysfunctional after hypoxia/ischemia [25–28] and that a TJ-barrier is formed early in development has been shown in a range of mammals including humans [29–32].

Given that TJ proteins have been suggested as potential biomarkers in adult brain injury models, we hypothesised that BBB related proteins could be detected in blood and cerebrospinal fluid (CSF) after neonatal HI. In this study we focused on three of the key TJ-proteins in the brain endothelium, claudin-5 (CLDN5), occludin (OCLN) and zonula occludens protein 1 (ZO-1). CLDN5 is an integral transmembrane TJ protein expressed by brain vascular endothelial cells adhering neighbouring cells together and sealing up the paracellular space between the cells of the BBB [33] with structural support from the intracellular scaffold protein ZO-1 [34] while OCLN acts as a regulator of TJ-function and remodelling [35]. In order to study the role of TJ proteins in neonatal brain injury we used a well-established model wherein a HI brain-injury is induced in post-natal day (PND) 7 rats by ligation of the left common carotid artery combined with a period of hypoxia [36], the brains of PND 7 rats approximates near-term human infants [37]. This model has been developed to approximate cerebral hypoxia–ischemia believed to be part of the aetiology of infants affected by NE [38]. Circulating TJ-proteins in blood plasma and cerebrospinal fluid (CSF), brain injury and BBB function after injury were determined.

## Methods

### Animals

Postnatal day 7 (PND7) Wistar rat pups were bred in-house at the Laboratory for Experimental Biomedicine of Gothenburg University (parents were sourced from Janvier Labs, Le Genest-Saint-Isle, France) and maintained under normal housing conditions with a 12 h light/dark cycle and free access to water and standard laboratory fodder. Animals of both sexes and different litters were used for the experiments and care was taken to minimise the number of animals used and to maintain an even sex-balance in all experimental and control groups. All experiments were approved by the Gothenburg Committee of the Swedish Animal Welfare Agency (Application nos. 663/17) and performed in accordance with the ARRIVE guidelines. A total of 104 animals were used throughout the study.

### Hypoxia–ischemia (HI)

PND7 rats were anaesthetised with isoflurane (3.5–5%, Vetmedic, Stockholm, Sweden) in a 50/50 oxygen/nitrogen mixture, placed on their backs and a small incision was made in the neck to gain access to the carotid artery. A suture was placed permanently around the left carotid artery and the incision sealed with Vetbond tissue adhesive (3 M, MN, USA). Surgery typically lasted for 3–5 min. Following surgery, pups were allowed to recover in their home cage together with their mother for 1 h. Subsequently, operated pups were placed in a 36 °C chamber. The chamber was perfused first with humidified air for 10 min followed by 8% oxygen for 1 h and then by humidified air for 10 min. After the hypoxic exposure, pups were returned to their home cages. Control animals was subjected to sham-surgery (anaesthesia and incision) but no hypoxia. During all procedures, animals were monitored for vitals (i.e. breathing and skin-colour); every animal in the study survived the surgery and hypoxia.

### Sample collection and processing

For all time-points after HI (i.e. 6 h, 24 h and 5 days) injured and control animals were euthanised with a lethal overdose of pentobarbital. Cerebrospinal fluid was collected from the cisterna magna through glass capillaries as described previously [39] and blood was collected by cardiac puncture with ethylenediaminetetraacetic acid (EDTA)-treated syringes. CSF was checked for blood contamination as previously described [40] and samples discarded when contamination detected (detection limit about 0.2%). Blood samples were centrifuged at 2000x*g* for five min to separate the plasma. Samples were placed on dry ice after collection and long-term stored in - 80 °C freezer until analysed. Whole brains (excluding the cerebellum and brain stem) were collected and immersed in cold 6% buffered formaldehyde (Histofix; Histolab, Gothenburg, Sweden) at 4 °C for 24 h before processing for paraffin embedding.

### Caspase-3 activity assay

The activity of cleaved caspase-3 at 6 h (n = 7) and 24 h (n = 7) after HI was measured using a fluorometric assay based on an earlier study [41]. Whole brain hemispheres where homogenised in cold RNase free phosphate-buffered saline (PBS) and sonicated in cold RNase free PBS containing 2% protease inhibitor cocktail (Sigma-Aldrich, MO, USA) and 10 mM EDTA. Aliquots were centrifuged at 10 000x*g* for 15 min in 4 °C and some supernatant were used for bicinchoninic acid concentration measurements. For caspase-3 activity, 20 μl supernatant were incubated with 80 µl extraction buffer composed of a buffer base (50 mM Tris, 100 mM NaCl, 5 nM EDTA, 1 mM egtazic acid, pH 7.3) and 0.2% 3-(3-cholamidopropyl)dimethylammonio-1-propanesulfonate, 1% protease inhibitor cocktail, and 1 mM phenylmethylsulfonyl fluoride (PMSF) (Sigma-Aldrich) on a 96 well plate for 15 min in room temperature (RT). 100 µl assay buffer made up of buffer base plus 4 mM dithiothreitol, 1 mM PMSF and 25 µM caspase-3 substrate (Peptides International, KY, USA) were added to the wells before the plate was read for 1 h at 37 °C with 2 min intervals on a SpectraMax Gemini EM microplate reader (Molecular Devices, CA, USA) set to excitation wavelength 380 nm and emission wavelength 460 nm. Endpoint readings were made before and after 10 µl of 10 µM free 7-amino-4-methylcoumarin (AMC) (Peptides International) and the V_max_ was calculated from the linear part of the curve, caspase-3 activity was expressed as pmol AMC/min·mg caspase-3.

### Enzyme-linked immunosorbent assay (ELISA)

Plasma- and CSF-samples were analysed using pre-coated ELISA kits for tight-junction proteins CLDN5 (Nordic BioSite. Stockholm, Sweden), OCLN (Cusabio, Wuhan, China), and ZO-1 (Cusabio) as per the manufacturer’s instructions. Plasma was diluted 20 times and CSF 10 times. In short, standards, CSF and plasma-samples were diluted in sample diluent buffer and incubated on ELISA-plates pre-coated with the antibody. After incubations with a biotinylated secondary antibody, horseradish peroxidase (HRP)-avidin, 3,3′,5,5′-Tetramethylbenzidine substrate, and a stop-solution the optical density was determined with a Spectramax Plus microplate reader (San Jose, CA, USA) set to 450 nm with 540 nm wavelength-correction (OCLN and ZO-1) or 450 nm (CLDN5). The protein concentration was determined from the resulting standard-curve. CSF and plasma from the same animals were analysed for both CLDN5 and OCLN, n = 7–8 for all time-points. Due to some differences between ELISA-plates, all data were normalised to the median of time-matched controls analysed on the same plate.

### Blood–brain barrier assessment

The blood–brain barrier permeability was measured using radiolabelled sucrose as described by our group earlier [39]. For all time points after HI; 6 h (n = 9), 24 h (n = 8), 5 days (n = 9), injured and PND7 and PND12 control animals (n = 6 for each age) were injected intraperitoneally (i.p.) with two µCi ^14^C‐sucrose (American Radiolabelled Chemicals, MO, USA) in saline (100 µl injection volume). Thirty min later, they were euthanised with a lethal overdose of pentobarbital. Blood was collected through cardiac puncture using a heparinised syringe and centrifuged at 2000x*g* for five min to separate plasma. Choroid plexuses were removed and whole cerebellum and brain stem as well as left and right hippocampus, cortex and striatum/thalamus were dissected and collected into pre-weighed scintillation vials and then re-weighed. 500 µl of Solvable (PerkinElmer, MA, USA) was added to all samples and they were incubated overnight in a 40 °C oven to dissolve the tissue. After checking that all tissues were solubilised, samples were left to cool down to RT, mixed with 10 mL Ultima Gold scintillation cocktail (PerkinElmer) and left for 60 min in darkness. The radioactivity in each sample was determined by liquid scintillation counting in a Tri-Carb 4910TR (PerkinElmer) and calculated as cpm/mg sample after background corrections. Brain/plasma sucrose concentration ratios were used as a measurement of BBB-permeability as previously described after correcting for residual blood space in brain [42]. These concentration ratios were calculated as a measure of BBB permeability in each region and ratios in the left (injured) hemisphere was compared to the right hemisphere as previously outlined [15].

BBB-disruption after HI was also tested using injections of Evans blue (EB) dye, a dye that binds to albumin in the blood and thus should be regarded as a high-molecular marker opposed to sucrose [43]. 4% EB dissolved in PBS were injected i.p. (4 µl per g body weight) 6 h post-HI (n = 3) and in control animals (n = 3). After 1 h, animals were euthanised with a lethal overdose of pentobarbital and transcardially perfused with saline and 6% buffered formaldehyde. Whole brains (excluding the cerebellum and brain stem) were collected and immersed in cold 6% buffered formaldehyde at 4 °C for 24 h before they were embedded in 4% agarose and cut in 100 µm thick sections in a Leica 1200 VT vibratome (Leica Biosystems, Wetzlar, Germany).

Sections were mounted in water-based CC/Mount (Sigma) and imaged at 680 nm, the wavelength in which emitted fluorescence from EB peaks [44]. EB-extravasation into the brain were quantified in cortical micrographs from injured animals (n = 3) and controls (n = 3). Images were segmented via thresholding, creating binary images of EB + -area which were measured and calculated as a percentage of the entire image area.

### Measurement of brain blood-vessel area

Entire hemispheres of CLDN5-stained fluorescent paraffin-sections of brains collected five days after HI and controls (n = 5 per group) were imaged with a tiling and stitching function. Two levels (700 µm apart) at mid-hippocampal level were imaged per animal and analysed with an in-house developed macro for the Fiji-build [45] of ImageJ [46] that utilises difference of Gaussian to eliminate all background while preserving all vessel information to accurately measure the area of blood vessels in an image. Briefly, entire CLDN5-stained brain hemispheres were imaged in a fluorescent microscope (Additional file 1: Figure S1a); the process is shown in a smaller selection of the image (Additional file 1: Figure S1b) for clarity. A copy of the image was subjected to Gaussian blur with sigma = 10 (Additional file 1: Figure S1c). The blurred image was subtracted from the original and threshold applied with Fiji’s “analyse particle”-tool to filter out any eventual debris so only marked blood vessels remained (Additional file 1: Figure S1d). The resulting vessel-image were then superimposed on the original image (Additional file 1) to confirm accurate vessel labelling. Blood vessel area was quantified in both injured and uninjured hemispheres by first outlining a region of interest (ROI) delineating the entire cortex and hippocampus and measure the total tissue area. Then the area of marked blood vessels within the ROI was determined and the percentage of blood vessel area of the total area in the hemisphere was calculated. Averages were calculated from the two mid-hippocampal levels per animal. Investigators were blinded to treatment groups during analysis.

### Immunohistochemistry and microscopy

Paraffin-embedded brains were cut in seven µm thick coronal sections at six levels and 40 sections apart with a microtome, starting at what corresponds to approximately -2.5 mm from bregma in an adult rat. For 3, 3′-diaminobenzidine (DAB) immunohistochemistry (IHC), sections were deparaffinised by 30 min incubation at 65 °C followed by xylene, and decreasing gradients of ethanol (100% to 70%), and rinsed in dH_2_O. Antigens were retrieved by boiling in citric buffer (10 mM, pH 6) before endogenous peroxidases were blocked with 3% H_2_O_2_. Unspecific binding was blocked by incubating sections in serum-free protein block (Aglient Dako, CA, USA) for 1 h in room-temperature (RT) followed by 4 °C overnight incubation with primary antibodies (the used antibodies were directed against platelet endothelial cell adhesion molecule (CD31) microtubule- associated protein-2 (MAP-2), CLDN5, and OCLN, diluted in PBS/0.05% Tween20 (see Table 1). After incubation with the appropriate biotinylated secondary antibodies (Vector Laboratories CA, USA) for 1 h at RT, the staining was enhanced by treatment with Vectastain Elite ABC HRP kit (Vector Laboratories). Finally, sections were dehydrated in gradients of ethanol (70–100%) followed by xylene and mounting in Pertex xylene-based mounting media (Histolab). For fluorescent IHC; deparaffinization, antigen retrieval, blocking, and antibody-incubations were performed as described above before mounting with ProLong Gold Antifade with or without 4′,6-diamidino-2-phenylindole/DAPI (ThermoFisher, MA, USA). Between all staining steps, sections were washed three times with PBS/0.05% Tween20 (except for after blocking). DAB-stained sections were imaged and photographed with a BX60 microscope equipped with a TH4-200 light-source using the cellSens software (Olympus, Tokyo, Japan) and fluorescently stained sections were examined with a Zeiss Axio Imager.Z2 equipped with Colibri 7 LED-light-source and a MRc AcioCam using the ZEN Blue software (Zeiss, Oberkochen, Germany).

**Table 1:** The antibodies used for immunohistochemistry

Primary antibodies	Secondary antibodies	Manufacturer (primary antibody)
Mouse-anti-MAP2 (1/1000)	Biotinylated horse-anti-mouse (1/250)	Sigma-Aldrich, M4403
Mouse-anti-rat CLDN5 (1/1000)	Goat-anti-mouse AF 594 (1/250)	ThermoFisher, 4C3C2
Rat-anti-mouse CD31 (1/100)	Donkey-anti AF 594 (1/250)	BD Pharmingen, MEC 13.3

### Quantification of CLDN5 expression in entire brain hemispheres

CLDN5 immunorecativity was quantified in brightfield micrographs of entire brain sections from all time points after HI. For each image, separate ROI:s were drawn around the left and right hemisphere and the images were, similarly to the EB-studies above, segmented via thresholding into binary images with CLDN5-positive areas marked. CLDN5 immunoreactivity was calculated as a percentage of the entire hemisphere area.

### Brain injury and tight-junction protein level

To test correlation of tight-junction protein levels and degree of brain injury, brains, CSF and blood plasma were collected from HI-animals (n = 12) 24 post-HI. The plasma and CSF were analysed for CLDN5 and OCLN with ELISA as described above while the brains were embedded in paraffin and sectioned to assess the brain injury. Grey matter tissue loss in the injured hemisphere was determined in brightfield-micrographs of coronal brain-sections stained for the neuron- and dendrite-marker Microtuble-associated protein 2 (MAP2). The images were analysed in ImageJ by delineating regions of interests encompassing the entire injured or uninjured hemispheres and measuring the MAP2 positive immunoreactivity in each hemisphere by investigators blinded to which groups and animals the images belonged to. The percentage of tissue loss in each level were calculated from the MAP2-positive area with this formula: (MAP2_uninjured_ − MAP2_injured_)/MAP2_uninjured_ × 100 [47]. In all animals, the analysis was performed at six levels encompassing the entire brain and the mean tissue loss of all levels was used in the correlation analysis.

### Statistics and graphs

Statistical analyses were made using GraphPad Prism version 8.00 for Windows (GraphPad Software, CA, USA). We used one-way ANOVA with Dunnett’s multiple comparison test, and Pearson’s correlations. The Benjamin-Hochberg method (FDR 0.1) was used to control for multiple correction problems when multiple t-tests were conducted. Specific tests are stated in each Figure legend. Principal component analysis was made using Qlucore Omics explorer software (Lund, Sweden) where the built-in statistics module was used to test differences between sexes on variables (unpaired t-test). Images were processed in the Fiji build [45] of ImageJ [46], figures were designed in Affinity Photo and Designer (Serif Europe, West Bridgford, United Kingdom). The variance of the data in the text of the results-section is presented as mean ± SD.

## Results

### HI induced caspase-3 activation in the injured brain hemisphere

In this model of neonatal HI, the combination of left carotid artery ligation and global hypoxia produces brain injury and tissue loss in the left hemisphere [36]. To confirm injury in all animals the activity of caspase-3, a hallmark of apoptosis, was measured in homogenates from both injured (left) and uninjured (right) hemisphere of HI and control animals (Additional file 2). Virtually no caspase-3 activity was detected in either the uninjured hemispheres of HI-animals nor in hemispheres of the controls while a significant increase in caspase-3 activity was seen in the injured hemisphere of HI-animals 6 h after HI compared to the uninjured hemisphere (p = 0.0344) or control animals (p = 0.028). Caspase-3 activity was further increased in the left hemisphere at 24 h after HI compared to 6 h after HI (p = 0.0087), controls (p = 0.0027) as well as the uninjured hemisphere (p = 0.0060). The range of caspase activity for the 6 h and 24 h post-HI groups were 3–77 and 10–950 pmol AMC/min x mg protein, respectively.

### HI-injury resulted in time-dependent increased levels of circulating tight-junction proteins CLDN5 and OCLN in cerebrospinal fluid and plasma and ZO-1 in CSF

**Fig. 1:**
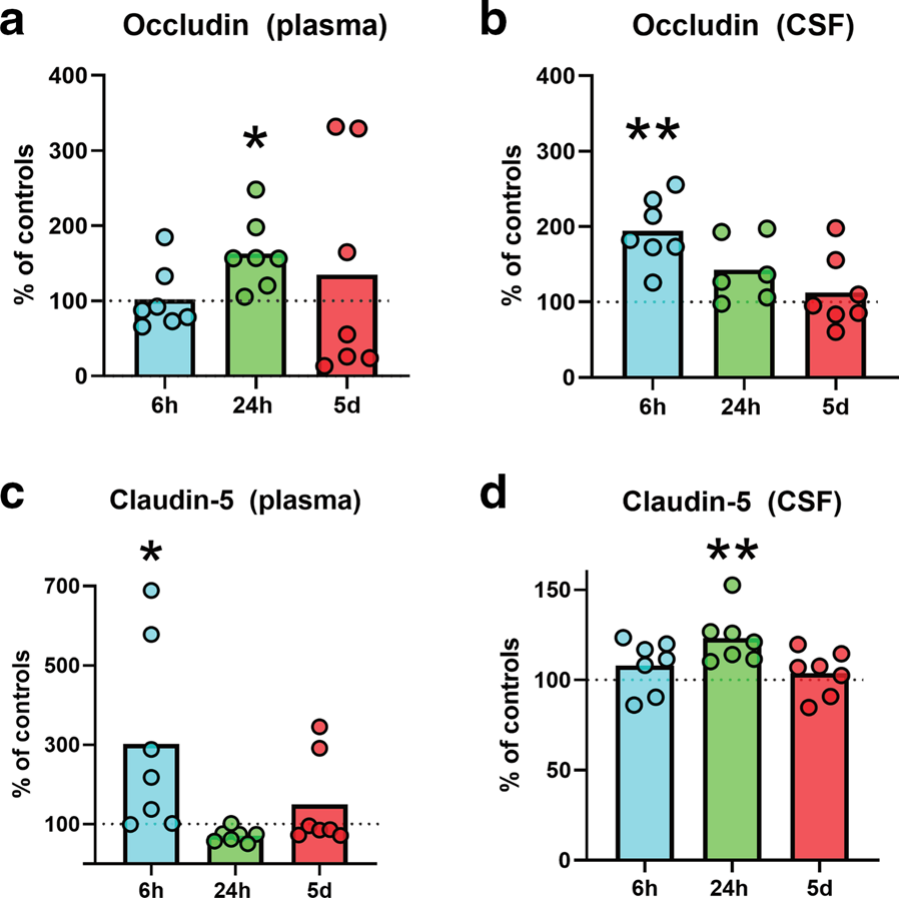
HI brain injury is followed by time-dependent increases of tight-junction proteins CLDN5 and OCLN levels in cerebrospinal fluid and blood plasma. The levels of TJ-proteins CLDN5 and OCLN were measured using ELISA in plasma (a and c) and CSF (b and d) collected at 6 h (3 m, 4f) and 24 h (3 m, 4f) as well as 5 days post-HI (4 m, 3f). Data is expressed as a percentage of the median in the corresponding control-groups (dotted line at 100%) and columns depict mean value, n = 7–8 per group. Unpaired t-tests using original concentration values, * = p ≤ 0.05 or ** = p ≤ 0.01. *m* male, *f  *female. Bar graphs represent mean values

Levels of CLDN5, OCLN and ZO-1 were measured in CSF and plasma with ELISA at 6, 24 h and 5 days after HI and all time-points were compared with a control group collected and analysed at the same time and on the same ELISA-plate. CLDN5 and OCLN proteins were detected in all samples with levels ranging between ~ 41 to 1300 pg/ml (plasma), ~ 100 to 2400 pg/ml (CSF) for OCLN and ~ 2 to 30 ng/ml (plasma), ~ 30 to 53 ng/ml (CSF) for CLDN5. Elevated CLDN5-levels were detected in CSF (Fig. 1d) at 24 h post-HI (p = 0.0082), while plasma-concentrations (Fig. 1c) were higher than controls at 6 h (p = 0.0427). There were no difference at later times between HI and controls. Similarly, OCLN concentration in CSF (Fig. 1b) was raised at 6 h (p = 0.0026) after HI while the levels were higher in plasma (Fig. 1a) of HI-animals at 24 h (p = 0.0285). Measured values (mean ± SD) for CLDN5 and OCLN in blood plasma and CSF are available in Additional file 3. ZO-1 was only detectable in 47% of CSF samples. We found great variably in levels between plasma with as high as 2000 pg/mL in one animal but no differences were measured across groups at any time point after HI (Additional file 4).

**Fig. 2:**
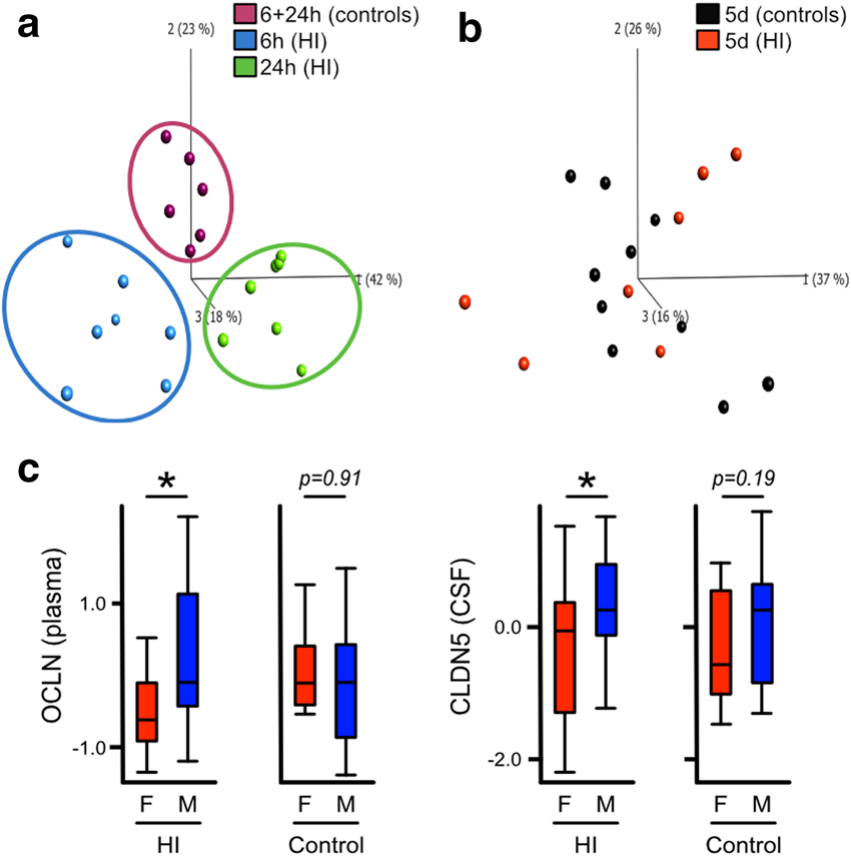
Principal component analysis discriminate between HI and control animals. Discriminate analysis (principal component analysis-plots) using CLDN5- and OCLN-measurements in plasma and CSF at different times after HI. Plots include 6 h (n = 7) and 24 h HI (n = 7) as well as 6 and 24 h controls (n = 6) which groups in three distinct groups in the analysis (**a**), this grouping in lost when analysing 5 days after HI (n = 7) and 5 days controls (n = 10) (**b**). Pooling all samples and adjusted for time as a factor (**c**) male rats (n = 10) were shown to have significantly higher levels of OCLN in plasma (p = 0.035) and CLDN5 in CSF (p = 0.036) than female rats (n = 11). No sex-difference were seen in plasma OCLN (p = 0.91) or CSF CLDN5 (p = 0.19) in control animals (males n = 7, females n = 9). Unpaired t-test, box-plots showing median, quartiles, and range (whiskers)

Principal component analysis (PCA) was performed (dimension reducing) as means of discriminate analysis with the input of all above measured variables for OCLN and CLDN5. HI and control animals were grouped into three distinct groups at 6 h and 24 h after HI (Fig. 2a) whereas at 5 days post-HI (Fig. 2b), no clustering of control vs HI animals was apparent. To test effect of sex on levels of TJ-proteins we performed unpaired t-test between male and female animals pooling all samples within all injured and within all control animals, adjusting for time as a factor. Male rats (n = 10) showed significantly higher levels of OCLN in plasma (p = 0.035) and CLDN5 in CSF (p = 0.036) than female rats (n = 11), control animals (males n = 7, females n = 9) had no sex-differences in plasma OCLN (p = 0.91) or CSF CLDN5 (p = 0.19) (Fig. 2c). In HI-animals, the average amount of OCLN in plasma were ~ 700 pg/ml for males and ~ 400 pg/ml for females, for CLDN5 in CSF the numbers were ~ 36 ng/ml for males and ~ 26 ng/ml for females.

### Dynamic changes in BBB function following HI in neonates

**Fig. 3:**
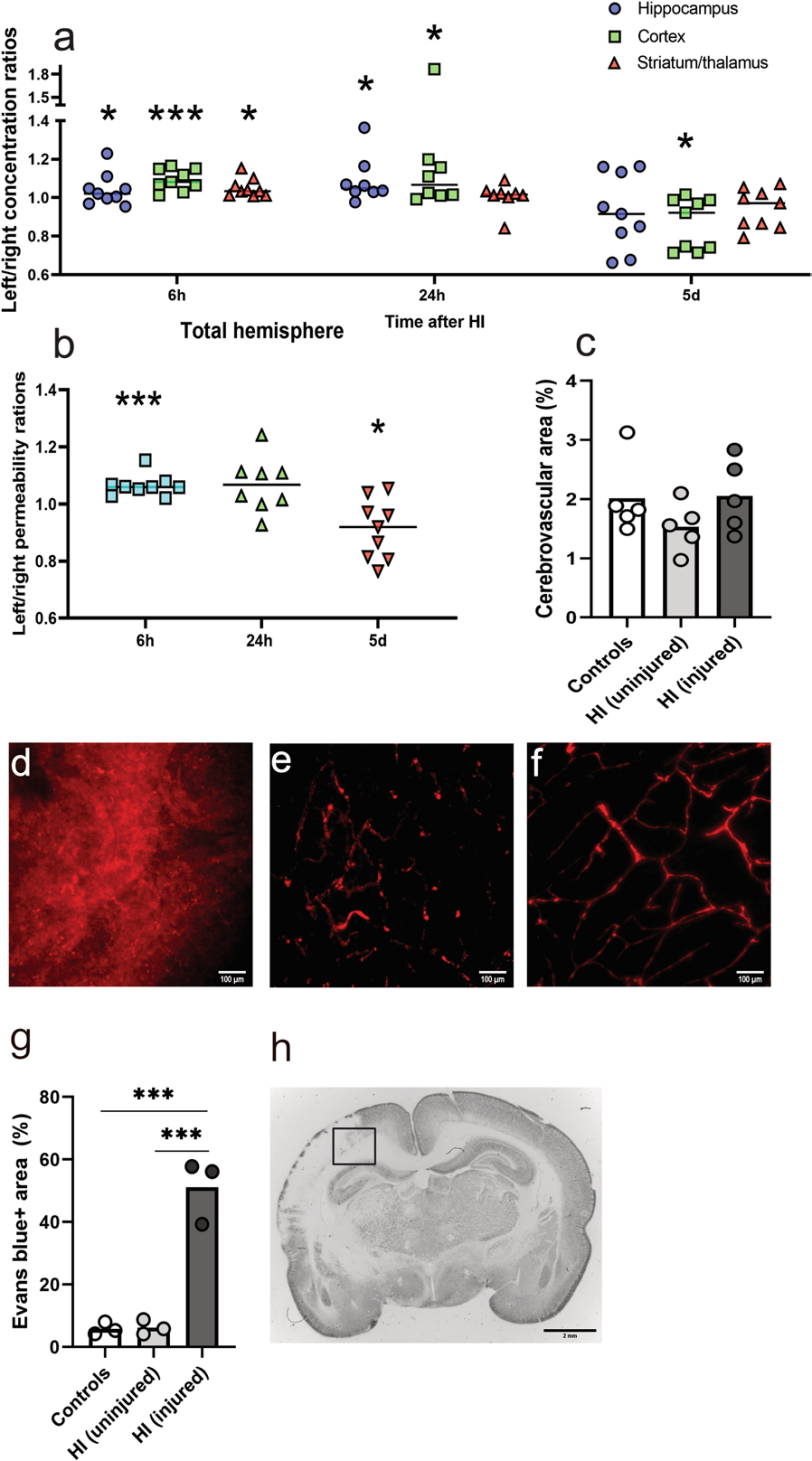
The BBB permeability increases in the injured hemisphere at 6 and 24 h after neonatal HI. **a** Regional (hippocampus, cortex, striatum/thalamus) brain ^14^C-sucrose concentration ratios between left (injured) and right (uninjured) hemispheres at 6 (4 m, 5f), 24 (4 m, 4f) hours and 5 days (4 m, 5f) after neonatal HI. **b** Entire hemisphere brain ^14^C-sucrose concentration ratios between left (injured) and right (uninjured) hemispheres at various times between 6 h and 5 days after neonatal HI. Horizontal lines indicate mean value. * = p ≤ 0.05, ** = p ≤ 0.01, and *** = p ≤ 0.001 (paired t-test between hemispheres). **c** The vascular density of the brain is not altered at 5 days after hypoxia/ischemia in neonates. Vascular area defined as CLDN5^+^ area in brain sections (See Additional file 1 for more details). Vascular area of total area in cortex/hippocampus in both the left (injured) and right hemispheres of HI-animals (n = 5, 2 m, 4f) was similar to control animals (n = 5, 2 m, 4f). BBB-damage were confirmed by imaging brain sections from animals injected with the fluorescent dye Evans blue (EB). After transcardial perfusion, areas of EB-bound albumin in the brain parenchyma was present in the cortex of injured hemispheres (**d**) while the uninjured hemispheres only showed weak EB-fluorescence restricted to blood vessels in corresponding areas (**e**). Control animals injected with EB and not perfused showed strong EB-fluorescence in blood vessels and none in parenchyma (**f**). Extravasated EB encompassed 51.1 ± 8.4% of the total area in images from the injured hemisphere, significantly altered from the uninjured hemisphere (6.3 ± 1.9%, p = 0.0002) and controls (5.8 ± 1.6%, p = 0.0002) where the EB was restricted to blood vessels (**g**), cortical images were were taken from the region outlined in (**h**). n=3 per group. Scale-bars are 100 μm (**d-f**) and two mm **(h**). One-way ANOVA with Dunnett’s multiple comparison test. Data presented as mean ± SD. *M* male, *f* female. Bar graphs represent mean values.

To determine changes in BBB function over time after HI we performed measurements of BBB-permeability in different brain regions (i.e. hippocampus, cortex, and striatum/thalamus) by quantifying the permeability for ^14^C-labelled sucrose across the BBB. We previously showed [15] that the BBB in the uninjured hemisphere is not altered. We confirmed this (Additional file 5) for the 6 h (controls n = 5, HI n = 5) and 5-day time-points (controls n = 5, HI n = 5) corroborating with caspase-3 activation (Additional file 2). Increased BBB permeability occurred in the ipsilateral hemisphere at 6 h post-HI in the cortex (1.10 ± 0.05, p = 0.0007), hippocampus (1.07 ± 0.08, p = 0.0277), and striatum/thalamus (1.05 ± 0.05, p = 0.0111) and was also significantly higher at 24 h in both hippocampus (1.12 ± 0.12, p = 0.0486) and cortex (1.18 ± 0.28, p = 0.0474) while the striatum/thalamus appeared unaltered (1.01 ± 0.07, p = 0.7624) (Fig. 3a). Mentionable is that these concentration ratios are probably somewhat affected by the edema occurring in the injured hemisphere after the insult suggesting that the magnitude of BBB permeability increase is likely marginally higher than what these ratios reflect. The concentration ratios 5d post-HI was significantly lower in the injured cortex (0.88 ± 0.12, p = 0.0191) as well as in the entire injured hemispheres (0.91 ± 0.10, p = 0.0303) (Fig. 3b).

BBB disruption following HI were confirmed via assessment of EB-bound albumin extravasation into the brain parenchyma in brain sections. Animals injected with EB at 6 h post-HI showed fluorescence in the cortex of the injured hemispheres (Fig. 3d) while the uninjured hemispheres showed weak fluorescence restricted to blood vessels (Fig. 3e). Control animals injected with EB and not perfused showed distinct blood vessels-restricted fluorescence (Fig. 3f) while animals not injected with EB showed no signal. Extravasated EB encompassed 51.1 ± 8.4% of the total area in images from the injured hemisphere, significantly compared to images from the uninjured hemisphere (6.3 ± 1.9%, p = 0.0002) and controls (5.8 ± 1.6%, p = 0.0002) where the EB was restricted to blood vessels.

### The vascular density of the brain is not altered five days after HI

Since the cerebrovascular area, the effective surface area for exchange between blood and brain, can affect measurements of BBB-permeability we developed an in-house written macro for blood vessel analysis of CLDN5 (as a vascular marker) immunolabelled sections. We specifically wanted to estimate cerebrovascular area at later times after injury since there is loss of brain tissue and potentially blood vessels. Sections from brains 5 days post-HI were used to calculate the area of blood vessels in the brain. Two levels were analysed per animal and results averaged (Fig. 3c). For this analysis, the hippocampus and cortex results were combined in each hemisphere. In control animals 2.01 ± 0.64% of the brain area was comprised of vessels while in HI animals vessels comprised 1.53 ± 0.37% of the uninjured hemisphere and 2.05 ± 0.61% in the injured hemisphere. No significant differences were detected between control animals and either hemisphere of injured animals (p > 0.05).

### CLDN5 immunoreactivity is not altered in the cerebral blood vessels of neonatal rats

**Fig. 4:**
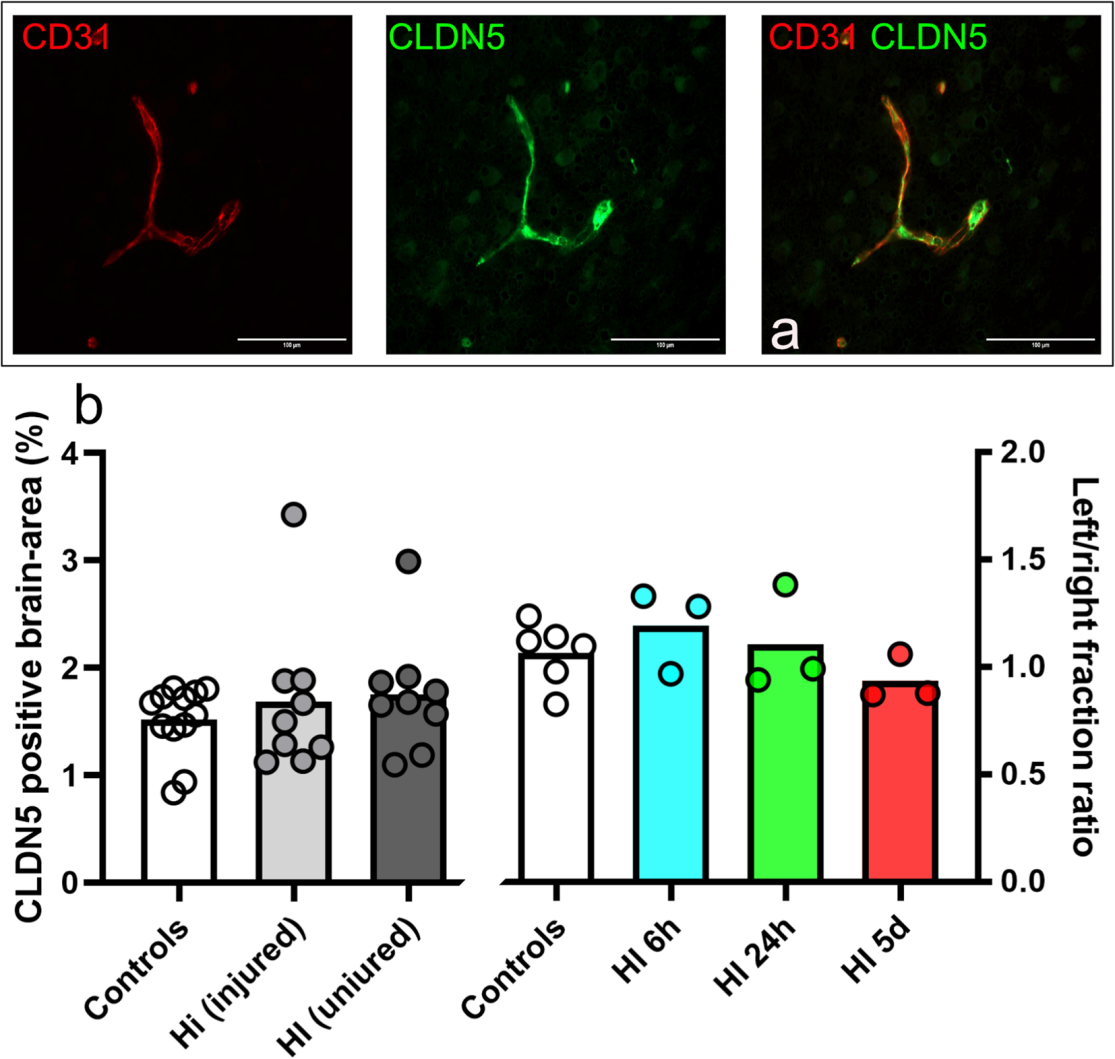
CLDN5 immunoreactivity is not altered in the cerebral blood vessels of neonatal rats after HI. Representative double-immunoreactivity for CD31 (red, blood vessel marker) and tight-junctional proteins CLDN5 (green) in cortical brain sections collected 6 h after hypoxia/ischemia (**a**). Scale-bars are 100 μm in all images. No difference in CLDN5-coverage were seen (**b**) between controls or either hemisphere in HI-animals at 6 h (2 m, 1f), 24 h (2 m, 1f), 5d (2 m, 1f) post-HI (left in b; p > 0.5) nor between the injured and uninjured hemisphere in HI animals (right in b; p > 0.05). HI, n = 3 per time point, controls (n = 2 per time-point). One-way ANOVA with Dunnett’s multiple comparison test and paired t-tests. *m*  male, *f* female. Bar graphs represent mean values

Given that there are reports of changes in TJ-protein immunoreactivity following hypoxia/ischemia and hypoxia alone [28, 48], we performed double immunofluorescent labelling of CLDN5 (Fig. 4a) together with blood vessel marker CD31 (platelet endothelial cell adhesion molecule) in paraffin brain-sections collected at all time points after HI (time chosen given BBB changes). In control animals, we found robust immunoreactivity of TJ-proteins in vessels in all brain regions examined including the cortex, hippocampus, and striatum/thalamus, while no labelling was detectable in parenchyma of the brain. Likewise, in animals after HI we found immunolabelling of blood vessels across all brain regions including MAP2-negative regions with no apparent changes compared to control animals. CLDN5 immunoreactivity was quantified in DAB-developed sections for CLDN5 only. No significant differences in CLDN5-coverage were seen between HI (n = 9) and control animals (n = 6) (p > 0.05), or between the injured and uninjured hemisphere in HI-animals (p > 0.05), at any time-point (n = 3 per time point) when CLDN5-immunoreactivity was quantified in entire stained brain hemispheres (Fig. 4b).

### CLDN5 levels in CSF correlates with brain-injury severity 24 h after HI

**Fig. 5:**
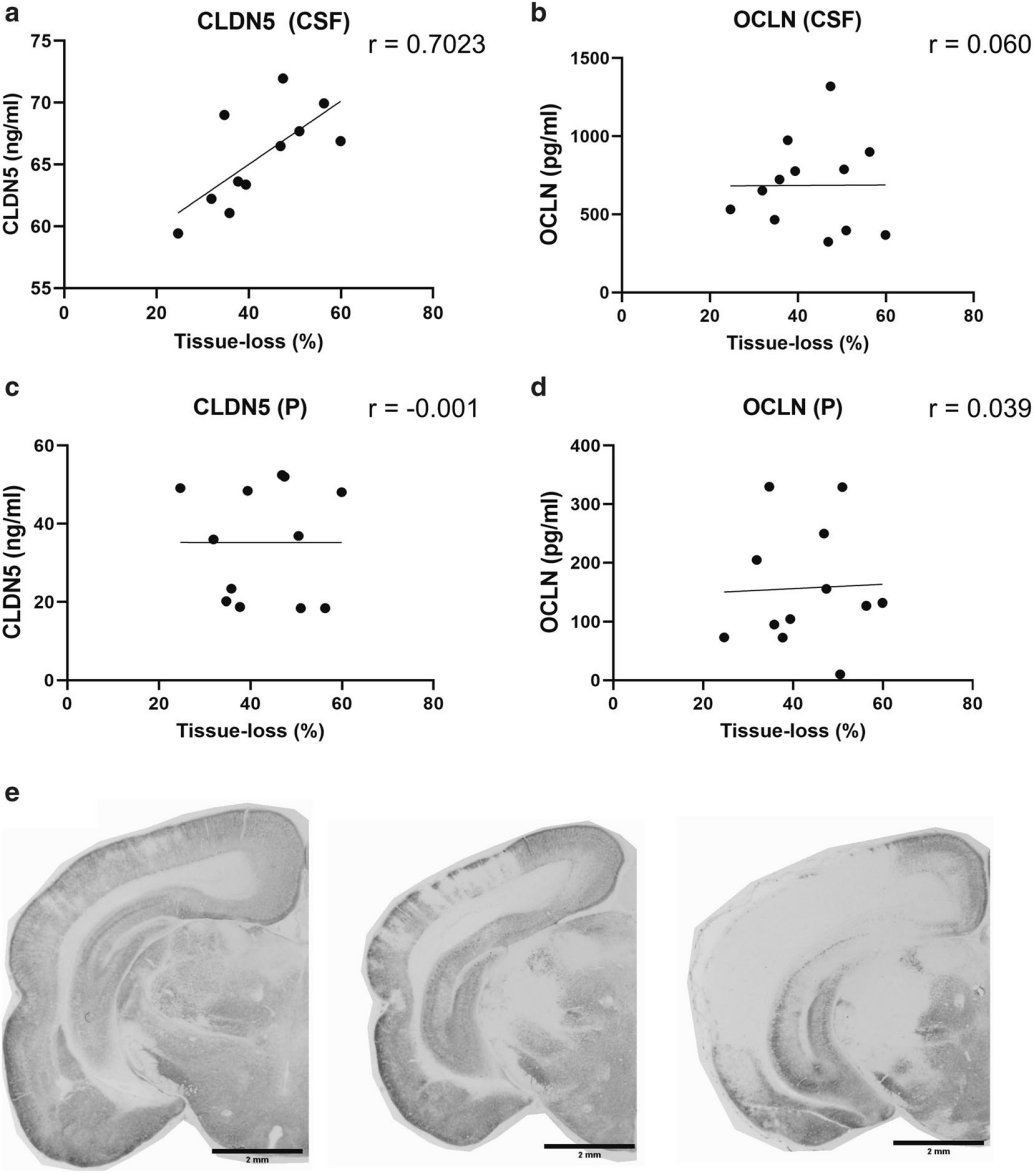
CLDN5 levels in CSF correlates with brain-injury severity 24 h after HI. The grey matter tissue loss, defined as the average percentage of negative MAP2 immunoreactivity calculated from six levels throughout the whole brain, correlates with the levels of CLDN5 in the CSF of the same animal 24 h post-HI (**a**) with a Pearson correlation coefficient (r) of 0.702 (p = 0.016). No correlation was observed between the loss of grey matter and levels of CLDN5 in plasma (**c**) nor OCLN in CSF (**b**) or plasma (**d**). The tissue loss varied between 24.7 to 59.9%, three representative images of injured brain hemispheres are shown in (**e**), n = 12 (5 m, 7f). Scale-bars are two mm in all images. *m*  male, *f * female

The correlation between circulating TJ-protein levels and brain injury severity were investigated 24 h after HI by analysing plasma- and CSF-levels of TJ:s and simultaneously quantifying the loss of grey matter in the brain of the same animal. This time-point was chosen based on the earlier ELISA-results (Fig. 1). The average brain tissue loss in the HI-group (n = 12) varied from 24.7 to 59.9% (one representative level is shown in Fig. 5b and d, respectively). By using the Pearson correlation on the levels of circulating TJ-proteins and the tissue loss percentages (Fig. 5a), the levels of CLDN5 in CSF was found to significantly (p = 0.016) correlate with the severity of the grey matter tissue loss (r = 0.702). No correlation was observed between the loss of grey matter and levels of CLDN5 in plasma (r = -− 0.001) nor OCLN in CSF (r = 0.060) or plasma (r = 0.039).

## Discussion and conclusions

The brain vasculature is a central component in HI associated brain injuries that appears damaged early in the injury process with studies indicating loss of BBB function in response to neonatal HI [15, 49]. However, diagnostic tools to evaluate brain vascular dysfunction in neonates in an early, affordable, accessible, and reliable manner are lacking. We show elevated levels of OCLN and CLDN5 in both CSF and plasma in response to loss of blood–brain barrier function and that CLDN5 levels in CSF correlates with brain-injury severity. Our data indicate that loss of BBB function is likely, at least partially, due to molecular impairment of endothelial tight-junctions after HI injury. The data suggest that the intermembrane TJ-proteins CLDN5 and OCLN are putative blood or CSF biomarkers for cerebral vascular dysfunction and brain damage in neonates.

The cerebrovasculature is specialised in that it harbours a range of barrier and transport mechanism not found in peripheral blood vessels, which compartmentalises the brain from the rest of the body so that brain cells can function in a controlled environment and provides protection for the brain from potential harmful blood solutes. These mechanisms are normally referred to as the blood–brain barrier and an essential part are the tight-junctions localised to the luminal side of the inter-endothelial cleft forming a physical barrier between blood plasma and brain [50]. The protein architecture of these junctions is complex with both inter- and intracellular elements. In this study we focused on two intracellular TJ-proteins, CLDN5, which are specific and essential for normal BBB function [51] and, OCLN, which plays a role in BBB modulation [52]. We made measurements of both BBB function together with measurements of TJ-proteins in both CSF and plasma in order to interpret data in an integrated manner.

We found time-dependent changes in both BBB function and TJ-proteins levels following HI. Detection of Evans blue in the injured region of HI-injured animals confirmed BBB-disruption following HI and the BBB permeability was acutely increased following HI in rat neonates in agreement with earlier rat studies [16, 20, 53] and with a similar time line to what we have seen in mice [15]. Clinical data show about five times higher albumin CSF/blood ratios of babies diagnosed with NE indicative of BBB damage also in human newborns, although such ratios should be interpreted with caution [49]. Overall, the greatest changes in BBB-opening appeared to be in the cortex, the region most affected in this HI-model of rats [54]. However, we also see a correlation between severity of injury and BBB opening in the different brain regions, similar to the earlier mouse study [15].

OCLN in CSF was significantly increased 6 h after HI while levels in plasma increased at 24 h. Levels of CLDN5 on the other hand were elevated in plasma at 6 h post-HI, while levels in CSF were higher than normal at 24 h. Levels of CLDN5 and OCLN appeared to be normalised in both plasma and CSF five days after HI. CLDN5 was more concentrated than OCLN in both plasma and CSF. High levels of ZO-1 could only be measured in the CSF in some of the animals and was not detectable in plasma, thus it appears that ZO-1, being an intracellular protein, is not as readily released into the blood after BBB damage. Ischemia has been shown to induce matrix metalloproteinase(MMP)-mediated disruption of TJ-proteins [55], thus altering the cellular distribution of CLDN5 and OCLN in neonatal mice [14] and adult rats [56] and seemingly releasing some TJ-proteins into the circulation.

Pan et al. [22] measured the levels of tight-junction proteins in blood up to 4.5 h after the induction of ischemic stroke via middle-cerebral artery occlusion (MCAO) in adult rats and saw a significant increase of circulating OCLN and a molecular loss of OCLN from cerebral microvessels at 4.5 h post-MCAO while reporting no differences in blood-CLDN5 levels. A follow up study yielded similar results in adult rats after MCAO as well as showing that blood OCLN was elevated in human stroke patients within 24 h and up to 3d after stroke onset, the levels of OCLN in blood was reduced after treatment with normobaric hyperoxia which inhibits MMP-9 activity [57]. While we cannot directly compare our results to their studies as they have utilised adult animals in another model, it is evident that focal stroke and MCAO leads to an increase in blood OCLN levels earlier than in our neonatal model where we see a peak in blood OCLN 24 h after HI.

The raised levels of TJ-proteins in both CSF and plasma indicate that BBB dysfunction after neonatal HI injury is likely to be at least partially due to direct molecular damage to the endothelial TJ:s. Intriguingly, the raised levels of circulating OCLN and CLDN5 did not occur at the same time after HI showing that these proteins although normally intimately localised do not appear to be released into these biofluids in the same manner. The exact cause of this unclear, but it has been shown that OCLN and CLDN5 are affected differently in the early ischemic stroke stages with the former being degraded by MMP:s and the latter instead redistributed via the membrane protein caveolin-1 [58]. The complex linking of CLDN5 and OCLN to domains of intercellular adapter proteins is different, as reviewed by Piontek et al. [59], and may also play a role in their release from tight-junctions.

Furthermore, we tested whether levels TJ proteins could reflect severity of injury, choosing the 24 h time-point since our results indicated particularly raised levels of CLDN5 in CSF at this time. This showed that levels of CLDN5 in CSF correlated with the severity of brain injury while OCLN levels and plasma CLDN5 showed no correlation. Human studies on circulating TJ proteins have only been made in adults, which makes direct comparisons with our results more difficult, but there are some common observations. Kazmierski et al. [23] measured TJ proteins in serum after ischemic stroke and found that levels of circulating CLDN5 and OCLN could predict clinical deterioration as a result of haemorrhagic transformation up to 4.5 h after stroke onset, with the most sensitive measurement being the CLDN5/ZO-1 ratio. Comparing TJ-levels in blood and CSF as well as BBB-disruption between controls and a cohort of patients with intracranial haemorrhage (ICH), Jiao et al. [24] showed that CSF, but not serum, levels of TJ proteins are sensitive predictors for BBB-damage after ICH. These studies and our results implies that the levels of circulating TJ-proteins indeed corresponds to the severity of vascular and tissue injury, and BBB-disruption following ischemic events in the brain. Their results also agree with our finding that circulating CLDN5 was more abundant than OCLN.

Taken together our study shows that even a moderate opening of BBB in this model results in raised levels of TJ-proteins at early time points after HI which suggests that TJ-proteins are released into the circulation in the early stages of BBB-damage and could act as biomarkers for vascular integrity and possibly also be useful as brain injury predictor. Our results also indicate a sex difference where males have higher levels of circulating OCLN in plasma and CLDN5 in CSF. This resonates well with previous studies that have shown that there is a tendency for male rats to have graver injuries than females after neonatal HI in an almost identical model to the one employed in this study [60] and it is also known that male human infants have a higher risk for NE [61]. Since our study was not specifically designed to investigate sex differences and analysis involved pooling samples from different time-points, further studies would be needed for confirmation of results. CLDN5 and OCLN showed different patterns of release into biofluids indicating that measuring both of them in tandem would give a better interpretation of injury to the brain vasculature after HI. We therefore performed discriminate analysis showing that HI-animals grouped together and were separate from controls up to 24 h after HI when the levels of CLDN5 and OCLN in both plasma and CSF were analysed together.

While TJ proteins seem to have potential as standalone biomarkers for neonatal cerebral vasculature dysfuntion, many studies, as reviewed in Douglas-Escobar and Weiss [62], Chalak [63], and Lv [13], have focused on biomarkers related to inflammation and brain injury. As the reviews state, the best course of action will most likely be to develop a panel of different markers in conjunction with diagnosis and other monitoring methods of neonatal brain injuries. Promising biomarker-candidates for assessing NE brain injuries include neuronal injury markers such as Tau and neurofilament light proteins [64], brain injury marker protein S100B [65], and inflammation-related cytokines like IL-6 and IL-8 [66]. The levels of circulating TJ-proteins in blood from human stroke patients has been shown to positively correlate with the levels of S100B [23] and we saw a correlation between white matter tissue loss and the levels of CLDN5 in CSF. We therefore believe circulating TJ-proteins have promise as a marker for vascular dysfunction, which, in combination with markers for inflammation and injury, would increase the discriminatory, and predictive power of a marker panel for NE brain injuries.

Intriguingly, our results indicated that BBB function does not normalise at later times after HI but instead there is an apparent decrease in permeability below normal levels five days after the insult. One explanation could be that there is loss of blood vessels in the brain tissue after HI, which would reduce the surface area for exchange and thus reduce flux between blood and brain. We therefore estimated the cerebrovascular area at 5 days after HI, which showed that the tissue remaining in the injured hemisphere did not differ in vascular area compared to control animals. Thus, loss of blood vessels unlikely explains the difference in measured BBB permeability. We previously demonstrated an upregulation of both CLDN5 and OCLN genes following neonatal HI injury in mice [15], which could be a response to normalise barrier function and might underlie the decrease in barrier permeability we observed in the present study. Brain blood flow, measured between 3 to 48 h, in the PND 7 rat HI-model have also shown that that cortical blood flow in this model is reduced not until 48 h after injury, indicating that HI could lead to alterations in the vascular physiology [67]. Speculatively, the time-dependent changes in BBB permeability, increased entry rate in the acute phase (6-24 h) followed by decreased entry rate later 5 days could influence the efficacy of drug treatment in relation to their time of administration time. A limitation of the study is the large inter-animal variability in injury, inherent to this model-system of neonatal HI [68]. Furthermore, brain injury is limited to the hypoxic/ischemic hemisphere, unlike human infants which often develop more generalised brain injuries [69].

## Impact statement

- BBB dysfunction following neonatal hypoxia/ischemia is likely in part due to the loss of tight-junction proteins from cerebral blood vessels. BBB breakdown release tight junction proteins and BBB function may be assessed by measuring these proteins in the circulation.
- This is the first study which investigates tight-junction proteins in the CSF and correlate to levels in circulation in a neonatal animal model of brain injury.
- Elevated levels of blood–brain barrier-derived tight-junction proteins Claudin-5 and Occludin can be detected in the circulation at several time-points in a rat-model for neonatal HI, signifying the proteins potential as biomarkers for the brain vascular dysfunction in neonates.

## Supplementary Information

**Additional file 1:** Images showing the process of delineating blood vessels from CLDN5 immunoreactivity in brain sections. Blood vessels in entire hemispheres (**a**) were delineated, the process is shown in a selection (**b**) for clarity. By utilizing difference of Gaussian (**c**) and Fiji’s “analyse particle”-tool, blood vessels in entire brain hemispheres were delineated (**d**). Accurate marking of vessels was confirmed by overlaying the processed image with the original non-processed image (**e**).

**Additional file 2:** Hypoxia/ischemia induces caspase-3 activation in the injured brain hemisphere only. Activated caspase-3 could be measured in the injured hemisphere 6h (3m, 4f), and 24h (3m, 4f) post-HI but not in the uninjured hemisphere nor in any hemisphere of control animals. Columns depict mean value, n = 7 per group. Unpaired t-tests between groups, significant differences are marked * (p ≤ 0.05) or ** (p ≤ 0.01).* m* male,* f* female. Bar graphs represent mean values.

**Additional file 3:** CLDN5 and OCLN levels in blood plasma and CSF, mean ± SD.

**Additional file 4:** Tight-junction protein ZO-1 can be detected in CSF from some, but not all, animals after neonatal hypoxia/ischemia. The levels of ZO-1 in CSF at 6h, and 24h as well as 5d after HI was measured with ELISA. Out of 7-8 animals per group, we could detect ZO-1 in 3-5 CSF samples. Columns depict mean value, one-way ANOVA with Dunnett’s multiple comparison test, HI-groups compared to the controls, p < 0.05 for all comparisons. Bar graphs represent mean values.

**Additional file 5:** BBB-permeability is not altered in right hemisphere of control animals. Blood/brain 14C-sucrose concentration ratios in the right hemispheres at 6h (3m, 2f) and 5 days (2m, 3f) following HI together with litter-mate controls. Horizontal lines indicate mean value, n=5 per group (mixed sexes), two-way ANOVA between regions and groups for both time-points, p<0.05 for all comparisons. .* m* male,* f* female.

## Data Availability

The data behind the conclusions of this study are available from the corresponding author upon reasonable request.

## Abbreviations

AMC: 7-Amino-4-methylcoumarin
BBB: Blood–brain barrier
CD31: Platelet endothelial cell adhesion molecule (CD31
CLDN5: Claudin-5
CSF: Cerebrospinal fluid
DAB: 3, 3′-Diaminobenzidine
EB: Evans blue
ELISA: Enzyme-linked immunosorbent assay
HI: Hypoxia/ischemia
HRP: Horseradish peroxidase
ICH: Intracranial haemorrhage
i.p: Intraperitoneal
IHC: Immunohistochemistry
MAP2: Microtubule Associated Protein 2
MAP-2: Microtubule- associated protein-2 (MAP-2)
NE: Neonatal encephalopathy
OCLN: Occludin
PBS: Phosphate-buffered saline
PCA: Principal component analysis
PMSF: Phenylmethylsulfonyl fluoride
PND#: Post-natal day #
TJ: Tight-junction(s)
ZO-1: Zonula occludens protein 1
