# Supplementary figures and images for: Circulating tight-junction proteins are potential biomarkers for blood–brain barrier function in a model of neonatal hypoxic/ischemic brain injury
Source: Fluids Barriers CNS. 2021 Feb 10;18:7. doi: 10.1186/s12987-021-00240-9 (PMC7877092; doi:10.1186/s12987-021-00240-9)

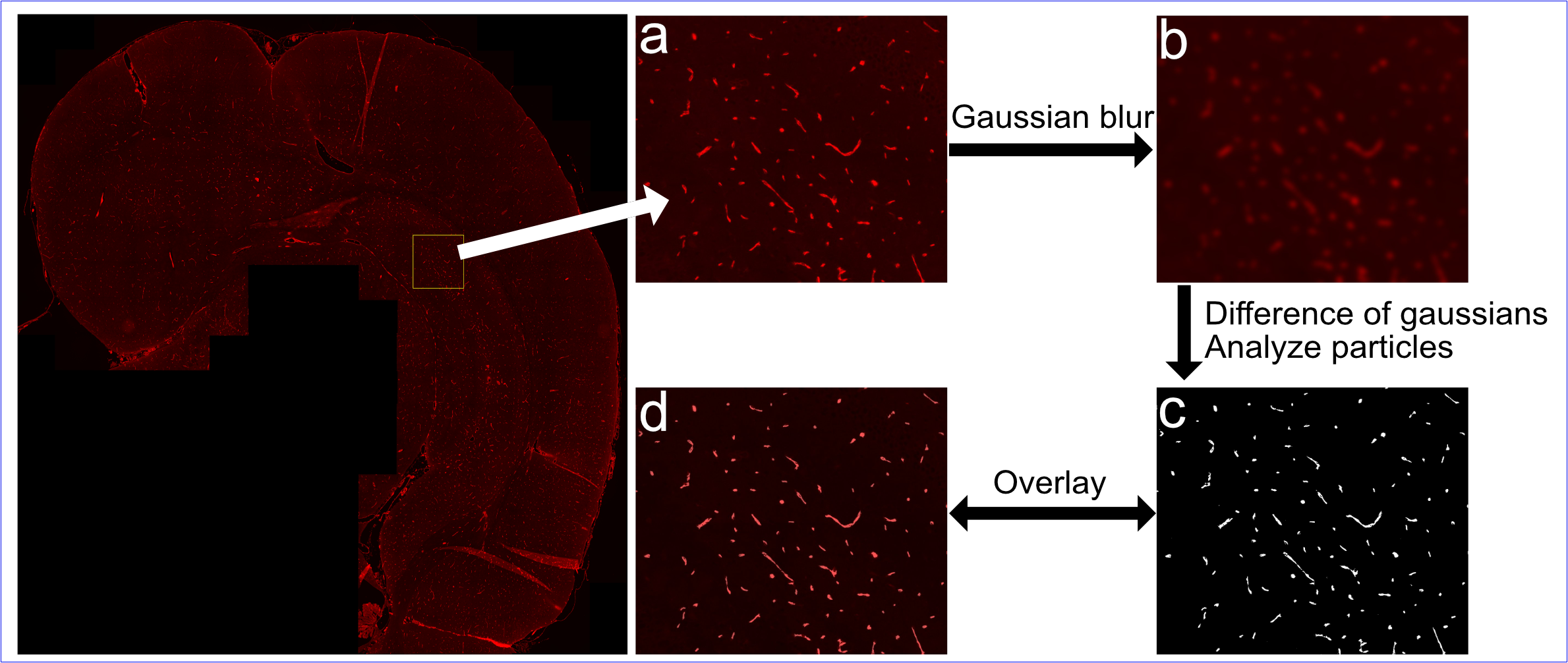

Supplement: Supplementary file 1 — Additional file 1: Images showing the process of delineating blood vessels from CLDN5 immunoreactivity in brain sections. Blood vessels in entire hemispheres (a) were delineated, the process is shown in a selection (b) for clarity. By utilizing difference of Gaussian (c) and Fiji’s “analyse particle”-tool, blood vessels in entire brain hemispheres were delineated (d). Accurate marking of vessels was confirmed by overlaying the processed image with the original non-processed image (e). [file 12987_2021_240_MOESM1_ESM.tiff]

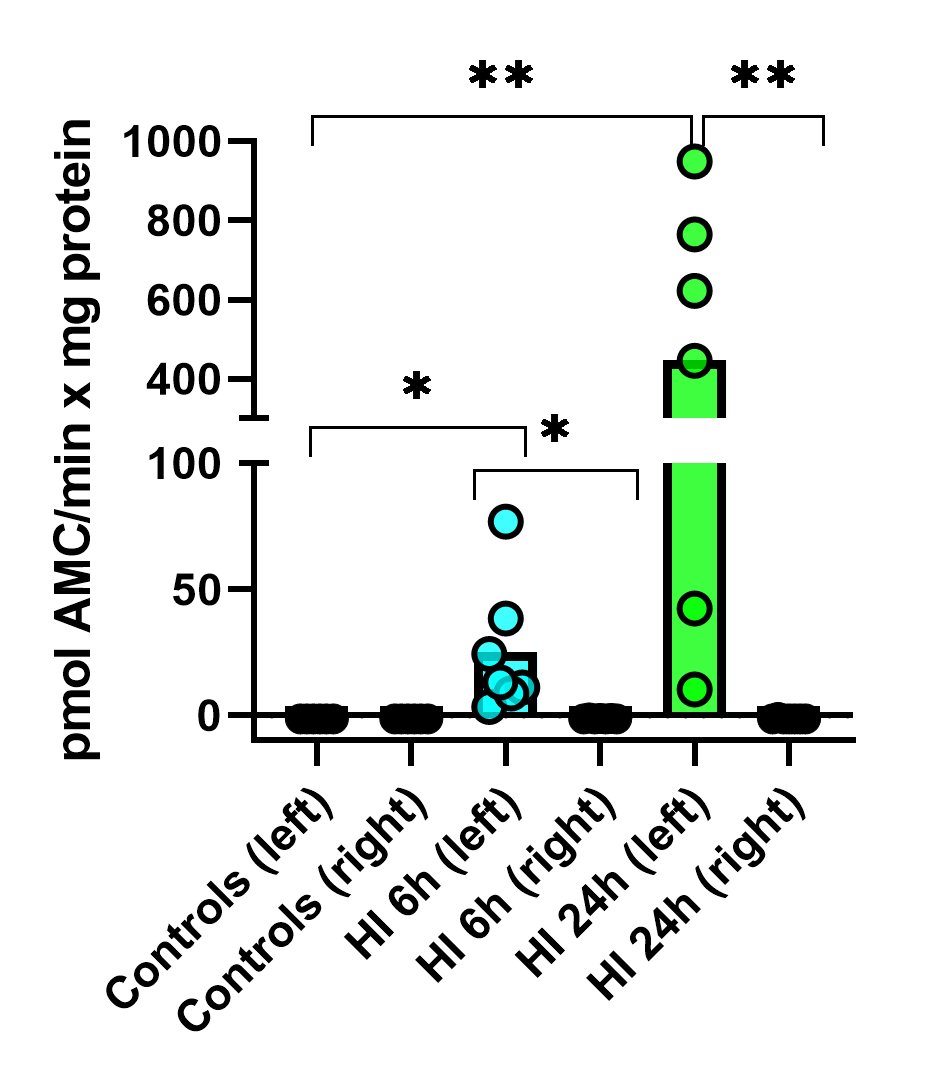

Supplement: Supplementary file 2 — Additional file 2: Hypoxia/ischemia induces caspase-3 activation in the injured brain hemisphere only. Activated caspase-3 could be measured in the injured hemisphere 6h (3m, 4f), and 24h (3m, 4f) post-HI but not in the uninjured hemisphere nor in any hemisphere of control animals. Columns depict mean value, n = 7 per group. Unpaired t-tests between groups, significant differences are marked * (p ≤ 0.05) or ** (p ≤ 0.01). m male, f female. Bar graphs represent mean values. [file 12987_2021_240_MOESM2_ESM.tif]

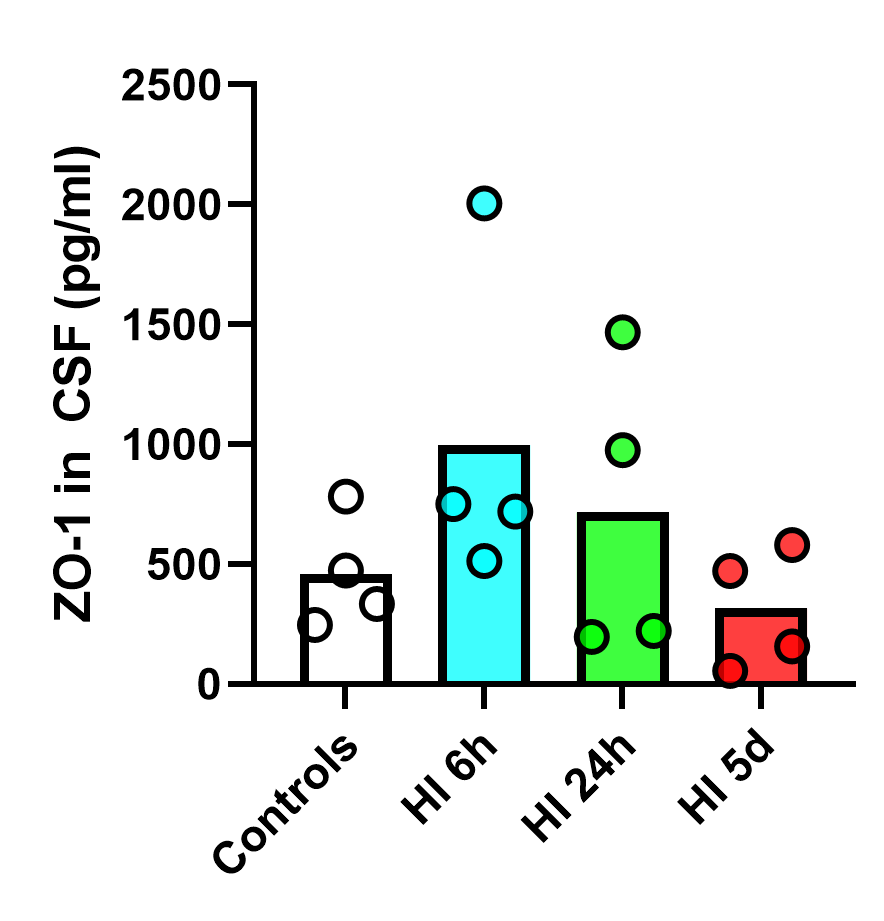

Supplement: Supplementary file 4 — Additional file 4: Tight-junction protein ZO-1 can be detected in CSF from some, but not all, animals after neonatal hypoxia/ischemia. The levels of ZO-1 in CSF at 6h, and 24h as well as 5d after HI was measured with ELISA. Out of 7-8 animals per group, we could detect ZO-1 in 3-5 CSF samples. Columns depict mean value, one-way ANOVA with Dunnett’s multiple comparison test, HI-groups compared to the controls, p < 0.05 for all comparisons. Bar graphs represent mean values. [file 12987_2021_240_MOESM4_ESM.tif]

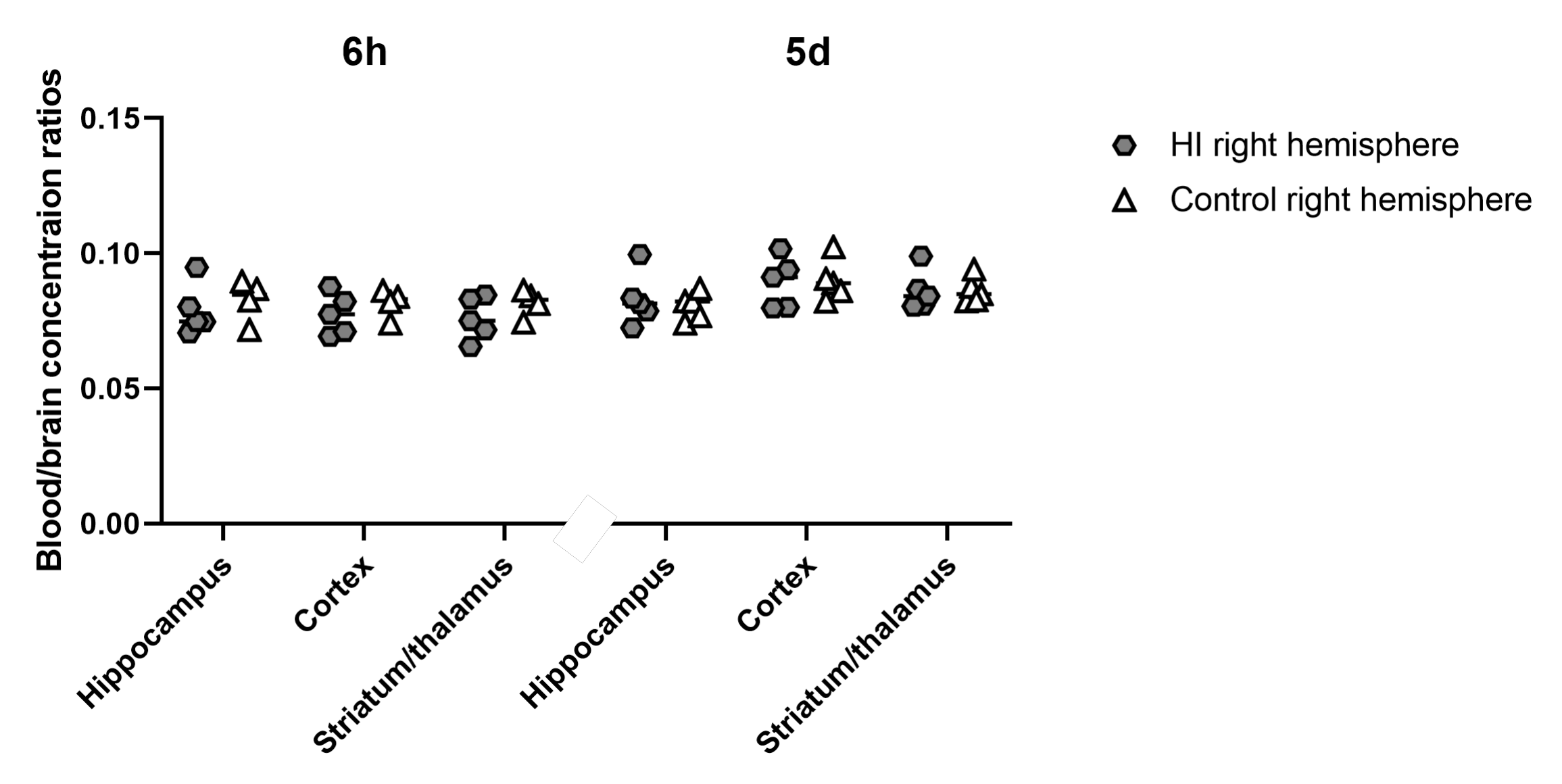

Supplement: Supplementary file 5 — Additional file 5: BBB-permeability is not altered in right hemisphere of control animals. Blood/brain 14C-sucrose concentration ratios in the right hemispheres at 6h (3m, 2f) and 5 days (2m, 3f) following HI together with litter-mate controls. Horizontal lines indicate mean value, n=5 per group (mixed sexes), two-way ANOVA between regions and groups for both time-points, p<0.05 for all comparisons. . m male, f female. [file 12987_2021_240_MOESM5_ESM.tiff]
