# Supplementary material for: Circulating tight-junction proteins are potential biomarkers for blood–brain barrier function in a model of neonatal hypoxic/ischemic brain injury
Source: Fluids Barriers CNS. 2021 Feb 10;18:7. doi: 10.1186/s12987-021-00240-9 (PMC7877092; doi:10.1186/s12987-021-00240-9)
Supplement: Supplementary file 3 — Additional file 3: CLDN5 and OCLN levels in blood plasma and CSF, mean ± SD. [file 12987_2021_240_MOESM3_ESM.docx]

**Additional file 3.** Tight-junction protein levels in blood plasma and CSF, mean ± SD

| Time-point | CLDN5 (plasma)  ng/ml | CLDN5 (CSF) ng/ml | OCLN (plasma) pg/ml | OCLN (CSF)  pg/ml |
| --- | --- | --- | --- | --- |
| Controls 6&24h | 3.98±1.04 | 34.60±1.17 | 388.29±59.03 | 173.83±97.95 |
| HI 6h | 11.53±8.44 | 37.18±4.61 | 378.70±145.62 | 400.35±83.94 |
| HI 24h | 2.71±0.59 | 2.71±0.59 | 604.93±164 | 284.56±78.39 |
| Controls 5d | 10.69±1.74 | 36.07±4.61 | 244.50±189.10 | 1245.74±826.42 |
| HI 5d | 15.86±11.44 | 36.05±4 | 413.52±405.92 | 1379.93±542.68 |
